# 3D image analysis reveals differences of CD30 positive cells and network formation in reactive and malignant human lymphoid tissue (classical Hodgkin Lymphoma)

**DOI:** 10.1371/journal.pone.0224156

**Published:** 2019-10-24

**Authors:** Julia Liebers, Patrick Wurzel, Kerstin Bianca Reisinger, Martin-Leo Hansmann

**Affiliations:** 1 Reference and Consultant Center of Lymph Node and Lymphoma Pathology at Dr. Senckenberg Institute for Pathology, Frankfurt/Main, Hessen, Germany; 2 Department of Molecular Bioinformatics, Johann Wolfgang Goethe-University Frankfurt/Main, Frankfurt/Main, Hessen, Germany; 3 Johann-Wolfgang-Goethe-Universität Frankfurt am Main, Frankfurt Institute for Advanced Studies (FIAS), Frankfurt/Main, Hessen, Germany; European Institute of Oncology, ITALY

## Abstract

**Aims:**

The examination of histological sections is still the gold standard in diagnostic pathology. Important histopathological diagnostic criteria are nuclear shapes and chromatin distribution as well as nucleus-cytoplasm relation and immunohistochemical properties of surface and intracellular proteins. The aim of this investigation was to evaluate the benefits and drawbacks of three-dimensional imaging of CD30^+^ cells in classical Hodgkin Lymphoma (cHL) in comparison to CD30^+^ lymphoid cells in reactive lymphoid tissues.

**Materials and results:**

Using immunoflourescence confocal microscopy and computer-based analysis, we compared CD30^+^ neoplastic cells in Nodular Sclerosis cHL (NScCHL), Mixed Cellularity cHL (MCcHL), with reactive CD30^+^ cells in Adenoids (AD) and Lymphadenitis (LAD). We confirmed that the percentage of CD30^+^ cell volume can be calculated. The amount in lymphadenitis was approx. 1.5%, in adenoids around 2%, in MCcHL up to 4,5% whereas the values for NScHL rose to more than 8% of the total cell cytoplasm. In addition, CD30^+^ tumour cells (HRS-cells) in cHL had larger volumes, and more protrusions compared to CD30^+^ reactive cells. Furthermore, the formation of large cell networks turned out to be a typical characteristic of NScHL.

**Conclusion:**

In contrast to 2D histology, 3D laser scanning offers a visualisation of complete cells, their network interaction and spatial distribution in the tissue. The possibility to differentiate cells in regards to volume, surface, shape, and cluster formation enables a new view on further diagnostic and biological questions. 3D includes an increased amount of information as a basis of bioinformatical calculations.

## Introduction

In diagnostic pathology, the examination of immunostained, thin tissue sections using a lightmicroscope is considered to be the standard [1]. Digital visualisation of these sections called Whole Slide Images (WSI), enabled diagnosis on computer screens and was extended by bioinformatic methods [2]. However, these approaches of tissue examination are confined to two dimensions [3–5].

Meanwhile, 3D visualisation of various tissue structures such as bones, vessels, soft tissue, various organs, etc. has become an integral part of diagnostics in clinical medicine, especially in radiology [6]. Such radiological approaches provide a deeper insight in human organ structures and enable a more accurate diagnosis as well as precise planning of operations, improved tumor radiation and therapeutic tracer application [7–10].

The resolution of two dimensional histological sections is usually superior compared to above-listed radiological methods [11]. It seems worthwhile to evaluate whether a 3D laser scanning approach of thick histological sections can add additional valid data to conventional histology, and in how far it might be superior to methods routinely used [12].

This investigation exemplarily focuses on a common malignant lymphoma in Europe, classical Hodgkin Lymphoma (cHL), especially its subtypes Nodular Sclerosis (NScHL) and Mixed Cellularity (MCcHL) [13]. We compare the typically CD30^+^ HRS-cells with reactive CD30^+^ large lymphoid cells, which are usually activated B-cells found in adenoids (AD) and lymphadenitis (LAD) [14].

Hodgkin lymphoma is derived from germinal center B-cells that clonally expand and have a non-functional B-cell receptor [15,16]. These genetically defective tumor cells pass immunosurveillance which subsequently ends in cell survival and tumor specific microenvironmental modulations. Thereby, T-cells and macrophages are predominantly attracted, leading to a massive enlargement of the lymph node [17,18]. Since the existence of HRS-cells is crucial for the diagnosis of cHL, the sole use of molecular biological methods, is inappropriate for diagnosis. Therefore, histological slices and microscopes still form an essential part in pathology. Furthermore morphology and immunohistochemistry, especially the high amounts of their characteristic surface marker CD30 defines cHL [13,17–20].

Differentiation between reactive and neoplastic CD30^+^ cells implies the necessity of a more precise definition of morphological features like cell shapes, surfaces, and interactions. This might be useful for prognostic and therapeutic evaluation, as well as for computer assisted diagnosis using bioinformatical techniques.

## Materials and methods

### Tissue preparation

Specimen samples originate from the archive of the Reference and Consultation Center of Lymph node and Lymphoma Pathology at the Dr. Senckenberg Institute of Pathology in Frankfurt am Main. Human adenoids were received from the Ear-Nose-Throat-center of the University hospital Frankfurt am Main after routine tonsillectomy.

All samples were collected between 2017 and 2018 and underwent anonymisation. The use of tissue samples was approved by the institutional guidelines of the Johann-Wolfgang-Goethe-University/Frankfurt as they cannot be related to any individual person.

Four groups of specimens can be distinguished: Adenoids (AD) (n = 10), lymphadenitis (LAD) (n = 10), Nodular Sclerosis classical Hodgkin Lymphoma (NScHL) (n = 10), and Mixed Cellularity classical Hodgkin Lymphoma (MCcHL) (n = 11). In MCcHL five cases of Epstein-Barr-Virus infected specimens are included.

For confocal microscopy formalin fixed samples, embedded in paraffin were cut into slices (18–33μm) using a microtome. Afterwards we deparaffined sections for 10 minutes in a xylene bath. For Rehydrating descending ethanol series were used before incubating the slices twice in 100% ethanol at room temperature. Then, slices were incubated twice for one minute in 96% ethanol. Afterwards with 70% ethanol for one minute, followed by aqua dest. for 5 minutes. Epitope retrieval was performed by cooking the loaded microscopy slides for 90 seconds in EDTA (pH 8, pressure cooker) before staining sections were transferred into aqua dest. Monoclonal Mouse, an Anti-Human CD30 Clone (Ber-H2, Agilent//Dako) served as primary antibody. As secondary antibody we used the VectaFlour R.T.U. Antibody Kit DyLight 594 including a normal horse serum (2.5%) for blocking and a goat anti-Mouse Ig amplifier antibody. Anti-goat IgG is the dye labeled immunoglobulin G. (Cat. No. DK-2594, Vector Laboratories, Burlingame, USA). Nucleic acid staining was performed with DAPI (D9542 Sigma Aldrich, St. Louis, USA). Vector TrueVIEW Quenching Kit (Burlingame, CA 94010) was used to minimise autofluorescence. Protocols were performed as recommended by the manufacturer.

Negative controls were prepared for each sample.

### Confocal imaging and processing software

All images were taken using a Leica SP8 Confocal microscope (Leica microsystems, Wetzlar, Germany) and under identical conditions. (Objective: HC PL APO 63x/1.3 GLYC CORR Cs2, Lasers: 405mm DMOD Compact, intensity: 1.1%; Green 552, intensity: 1.6%) Scan format was 1024x1024 pixels. Z-stack interval was 0.13mm. Image size was 365x375ym each to achieve an ideal compromise between acquisition time, image analysis and processing capacity.

Image analysis was performed with IMARIS Advanced Tracking 9.2 Software (Bitplane AG, Zurich, Switzerland).

We used the tool “Create surface” based on “Absolute Intensity” for calculating the surface of CD30^+^ cells with the automatic settings of IMARIS. CD30+ areas show the immunostained surface membrane and cytoplasm which surrounds the nucleus. The latter is stained with DAPI and is not included in the values. In the following, the term “volume” is used for these positively stained cell components in every case of our samples. For precise calculation, controls were scanned and the surface volume of small objects were subtracted from the final image in avoidance of artefact due to left antibody precipitates in tissue or nonspecific binding.

### Image analysis

**Fig 1:**
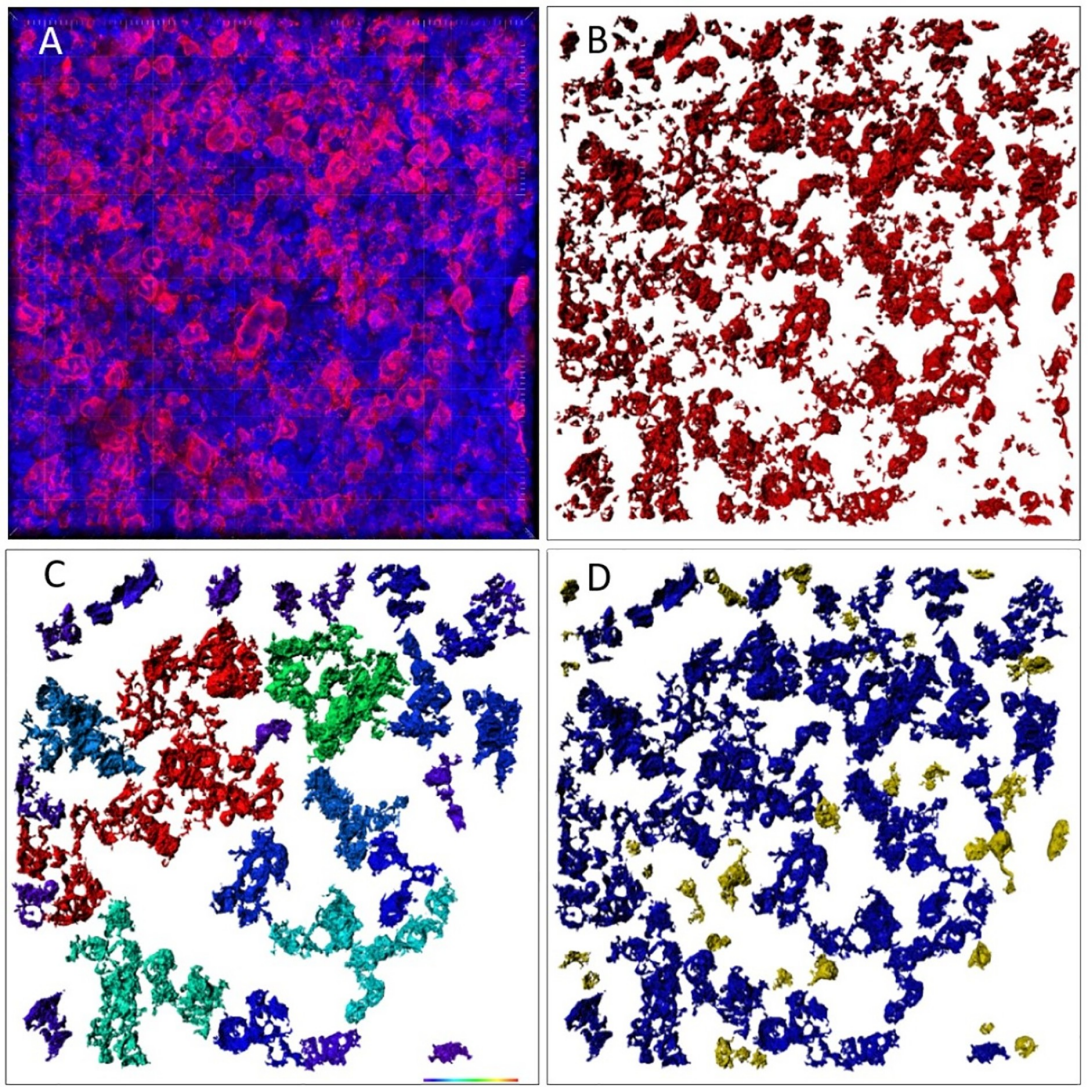
Example of the image analysis scheme. (A) A confocal image represents raw data. (B) Surface reconstruction of CD30^+^ cells and cell particles within IMARIS. (C) Cell networks coloured according to their size. (D) Single cells coloured in yellow.

Areas of high density of CD30^+^ cells were selected, scanned and surface creation was implemented. We differentiated between solitary cells (nearly no contact to others) and networks (cell aggregates of at least three cells). Fig 1 demonstrates the steps for image analysis. In order to obtain statistic data, we calculated absolute volume of cell cytoplasm, volume of network, and single cell parameters in reactive tissue and compared those to tumor cells. Statistics were performed with Python 3.6.1.

## Results

### Visual differentiation 2D and 3D

**Fig 2:**
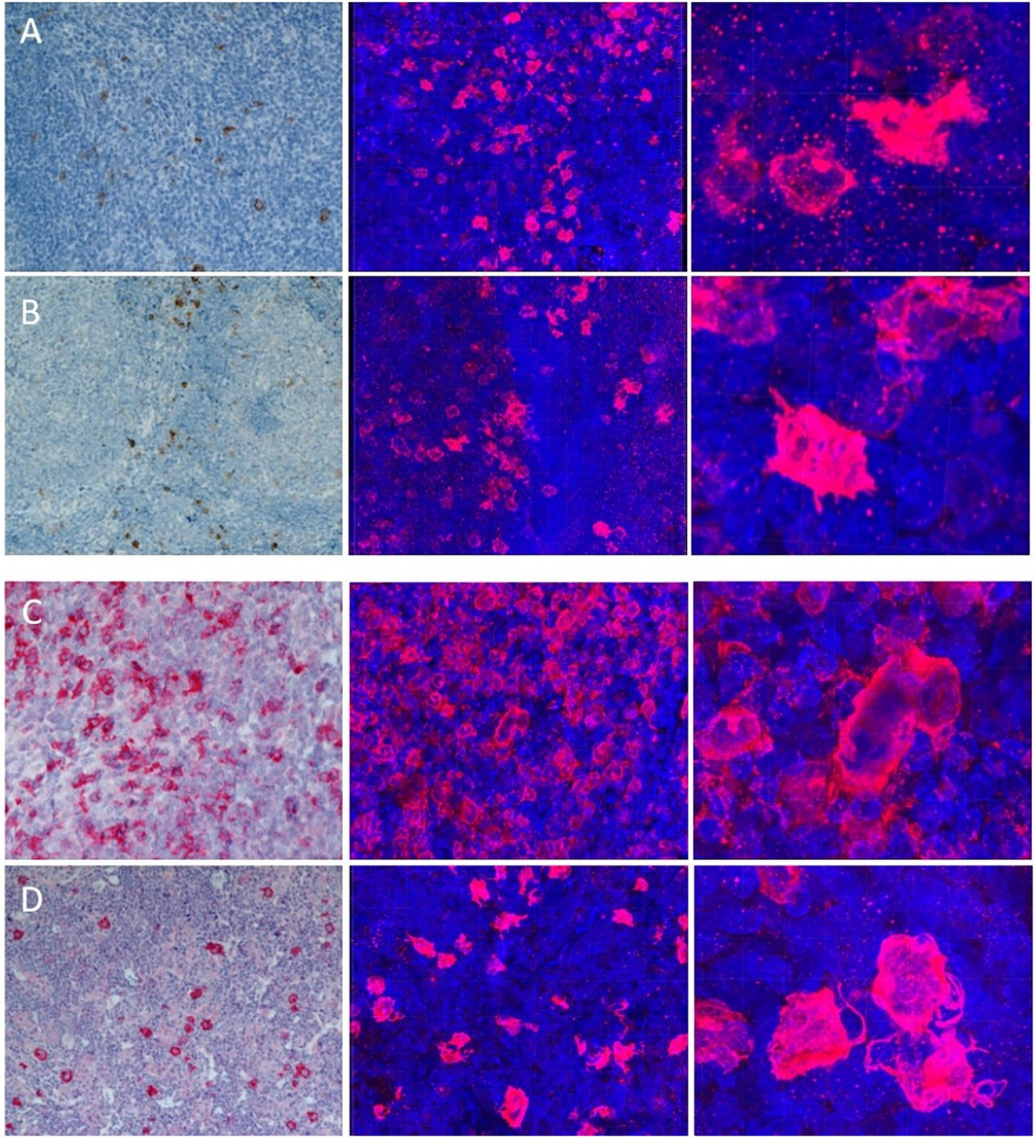
Comparison of conventional microscopy to confocal microscopy. **A** to **D** show: AD (A), LAD (B), NScHL(C), MCcHL (D) in conventional microscopy (10x magn.). Next to them the same entities visualised by a confocal microscope (20x magn.). Note the network formation in NscHL (C) and filigree protrusions in MCcHL (D) in the detailed image cutouts (40x magn.).

In all selected cases of reactive and neoplastic lymphoid tissue, CD30^+^ cells could be found. Fig 2 shows 2D and 3D images of the identical cases of each entity.

In conventional sections of the AD (Fig 2A) the CD30^+^ cells are predominantly located interfollicular (between germinal centres), in the mantle zone or at the border between follicular mantle and T-zone. CD30 is moderately expressed. Usually a few CD30^+^ cells are scattered in the germinal centres, being weakly positive for CD30.

In LAD (Fig 2B) the proportion of CD30^+^ cells vary from case to case. In most of the sections, CD30^+^ cells were immunostained and distributed similarly to those of the lymphoid tissue of the adenoids. In some cases, numerous strongly activated, positively stained CD30 lymphocytes can be found in the medulla of lymphadenitis.

Our cases of NScHL and MCcHL (Fig 2C and 2D) showed the typical histological characteristics for HRS-cells. In Nodular Sclerosis Hodgkin-cells and large HRS-cells formed syncytial clusters, whereas in MCcHL, rare or moderate amounts of HRS cells could be observed which were diffusely distributed in a background of reactive lymphocytes and histiocytes. In routinely cut 2D histological sections, protrusions of the HRS-cells seem to exist, but this cannot be confirmed in these thin sections.

A 3D image analysis allowed a precise characterisation of cell morphology considering its surface, cell or cytoplasmatic volume, and cell distribution in human tissue.

**Fig 3:**
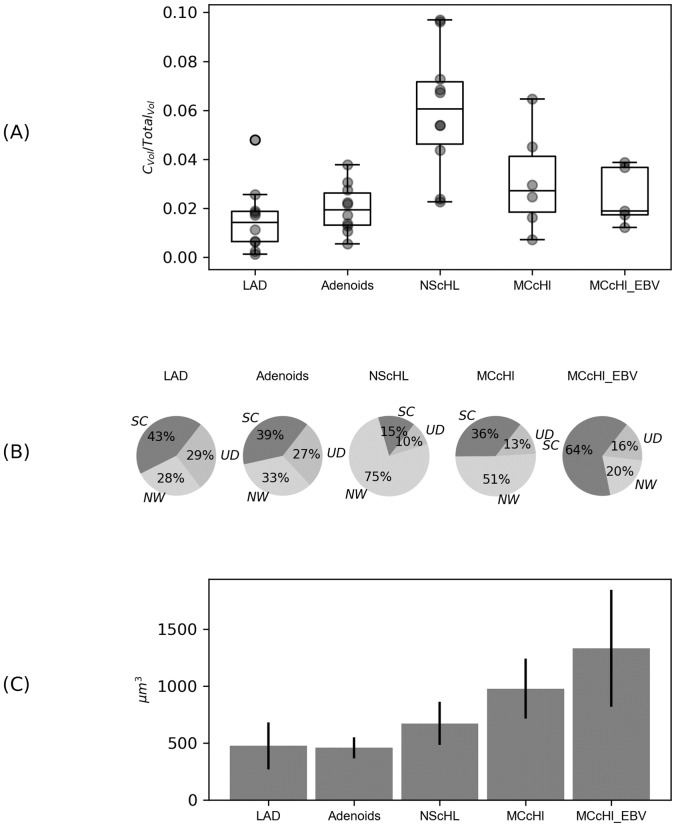
Comparison of CD30^+^ cell cytoplasm under reactive and neoplastic conditions. (A) displays the percentage of the total amount of CD30^+^ cell cytoplasm and cell particles in tissue sections of different entities. (B) illustrates the percentages of CD30^+^ cytoplasm of networks (NW), single cells (SC) and undefined (UD) in the total of CD30^+^ volume for each entity of all cases (41). UD were not definable surfaces which mean cell fragments which were located at the edge of the cutout. Reactive Adenoids and LAD show nearly equal distribution. NScHL is dominated by networks whereas in MCcHL most of the CD30^+^ volume is allocated to single cells. (C) depicts the values for the average stained volumes of these single cell cytoplasm in μm^3^. The difference in size of CD30^+^ cell cytoplasm varies in the four entities. Cell networks are excluded. LAD, lymphadenitis; Adenoids; NScHL, Nodular Sclerosis classical Hodgkin Lymphoma; MCcHL, Mixed Cellularity classical Hodgkin Lymphoma; MCcHL EBV^+^, with EBV infection.

The absolute volume of each imaged tissue section was calculated. By surface and volume reconstruction performed with IMARIS, the real amount of CD30^+^ cell cytoplasm was predictable. Fig 3 shows the visualised data.

They amounted to 1.51% (± 1.37) in LAD and to 1.99% (± 0.94) in AD. In malignant transformed entities, the value of HRS-cell volume was slightly higher, in particular 5.99% (± 2.45) in NScHL and in MCcHL EBV^-^ 3.13% (± 1.90) as well as in MCcHL EBV^+^ 2.23% (± 1.42).

Furthermore, the additional parameters as networks, single cells, cell shapes, intercellular contacts, and the presence of protrusions have been evaluated by 3D images. Table 1 shows parameters that can visibly distinguish between the entities by a 3D depiction, whereas the pie charts in Fig 3(B) show the cytoplasm values of single cells and the network proportions for each entity. In NScHL an extensive accumulation of Hodgkin and HRS-cells was observed which often form networks. Network formation was also visible in reactive conditions, however less frequent and in a lower extent. In MCcHL the size of the CD30^+^ cells and their round shape was a conspicuous feature, as well as their filigree protrusions.

**Table 1:** Additional parameters for morphological prescription of CD30^+^ cells in reactive (LAD, AD) and neoplastic (NScHL, MCcHL) entities.

Entity	LAD	AD	NScHL	MCcHL
**Intensity of CD30 Staining**	Variable, case-dependant	Variable, weaker in GC	Homogenous, mostly intense	Homogenous, Intense
**Networks**	Rarely	Often	Always	Rarely
**Network size**	Small	Medium	Large	Small
**Protrusions**	Existing, sparsely developed	Clearly pronounced	Existing, sparsely developed	Frequently distinct, occasionally filiform
**Cell shape**	predominantly round	Variable	predominantly round	round, large
**In contact to Others**	often	Often	mostly	barely

Fig 3(C) displays the absolute volumes of the cytoplasm of single cells of the four entities. Reactive entities shared similar cell volumes: 464 μm^3^ in LAD and 458μm^3^ in AD. The Standard deviation of ±207μm^3^ in LAD was twice as much as in AD (±92μm^3^). Single cells of NScHL were slightly larger with an average of 672μm^3^ (±190μm^3^). Largest volumes arose in the subtype MCcHL with 978 μm^3^ (±264μm^3^), especially when infected with EBV 1335μm^3^ (±515μm^3)^.

## Discussion

This paper shows a 3D investigation of human lymphoid tissue with focus on CD30^+^ reactive and neoplastic cells. Many papers on cHL claim that HRS-cells are below 1% in the infiltrates. However, it is unclear if the total amount of cells, or the percentage of cell surfaces of the microscopic slice are meant. The best parameter would be the volume of tumor cells compared to the volume of bystander cells which was difficult to measure until now, as conventional 2μm thin sections do not display entire cells. Furthermore serial-sections, which cause discontinuity of the tissue, lead to artifacts in the 3D image and often need to be rearranged manually in a time-consuming procedure [21].

In recent years laser scanning techniques were established that enable quick reconstruction of cell volumes, surfaces and intercellular connections using z-stacks with appropriate software.

Especially cell contacts play a crucial role in antigen presentation, cell activation and subsequent immune response. Our investigation focuses in particular on the transmission of histologic features of a 2D slice to a 3D depiction. We compare differences in intercellular connections of CD30^+^ cells in human lymphatic tissue under reactive and neoplastic conditions using paraffin embedded tissues as they are used for routine diagnostic.

**Fig 4:**
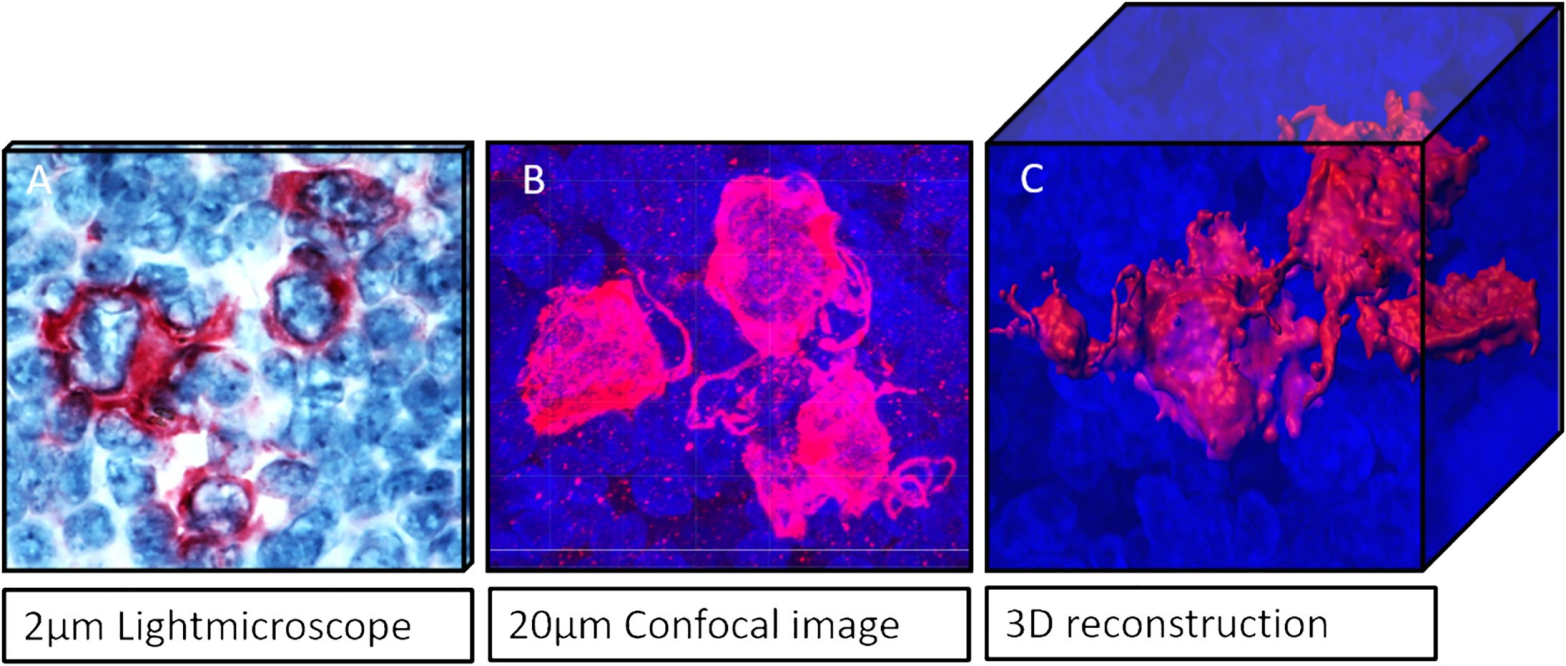
Comparison of imaging techniques in 2D and 3D. (A) conventional histological CD30 staining in MCcHL. (B) Representation of the same case in three dimensions using a confocal laser scanning microscope. (C) Surface reconstruction of the cell arrangement of a confocal image. The 3D image illustrates the spatial relationship of the cells to each other, their contacts, protrusions and contiguous structures. Images can be rotated in all three axes on the computer.

**Fig 5:**
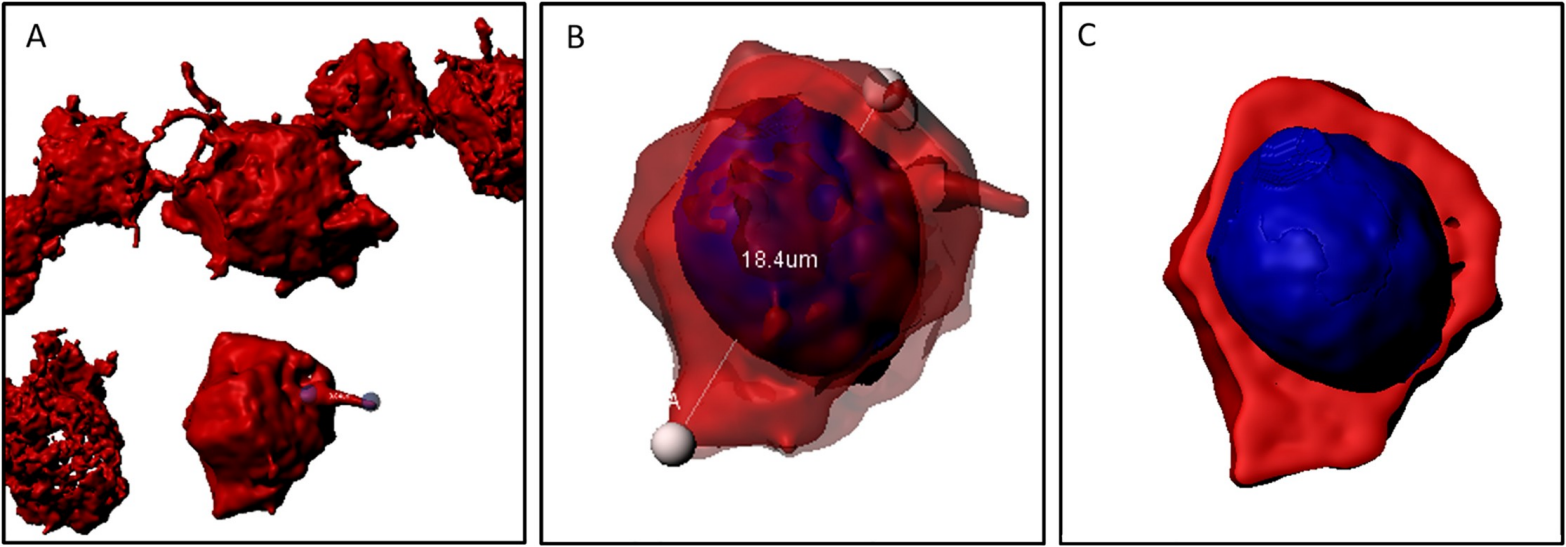
(A) sample of CD30^+^ cells of a MCcHL. CD30^+^ cells are connected by protrusions. (B) shows an excerpt of (A). The cell is semitransparent to determine its diameter (18,4μm) and to shows the nucleus colored in blue. (C) section through the cell. The cytoplasm and the nucleus can be distinguished from each other. The CD30^+^ proportion (cytoplasm) represents the share we used for calculations.

Fig 4 shows the impact of the 3D visualisation in comparison to 2D conventional diagnostic techniques. The transition from a 2μm thin light microscopic picture to a 3D reconstruction reveals morphological properties like surface, shape, volume, and cell connections. Since, 2D slices only represent one stage of a complex 3D cell system, cell protrusions cannot clearly be assigned to a particular cell. Furthermore, the overlapping of cells occurring in 2D slices can cause misinterpretation due to blurring of cell boundaries. 3D imaging even offers the ability to separate the nucleus from the cytoplasm, to rotate objects in three axis and to observe the slice view, analogous to the depiction to a conventional histological section. (Fig 5)

Advantages of confocal 3D imaging were recently exemplarily published on prostate epithelia lesions which revealed better visual differentiation than on 2D slices [22]. Furthermore studies on undefined malignant lymphoma and human lungs proved nuclear atypia in malignant transformed cells by using 3D imaging and clearing techniques [23]. However, to the best of our knowledge, there is no 3D investigation on lymphatic tissue especially cHL including parameters like volume, shape and networks for quantitative evaluation.

Recently, computer-analysis programs for cell dissection and recognition have been developed. Subsequently, data collected by computers can be obtained faster than by manual effort and evaluated automatically [6]. In cHL, automatic cell detection was performed by Schaefer et al., demonstrating that HRS-cells compared to reactive cases of LAD have a different vertex degree distribution and tend to form clusters [24]. Such image analysis pipelines, which were performed on 2D sections, may contribute to the computer-based diagnosis finding process in the future. Therefore, 3D approaches may improve the diagnostic accuracy in digital pathology [25]. Using the surface creation wizard, a tool for automatic cell recognition and surface reconstruction, we were able to reconstruct surfaced within seconds. Manually set thresholds can be used to optimize the visualisation.

In addition, stored data can be further processed and subsequently objects be printed to 3D models, as shown in the example of the networks of human fibroblasts [26]. 3D models may lead to a better communication between pathologists, students, clinicians and patients.

Analogous to conventional histology, the 3D visualisation gives information about a specific point in time of the tissue structure and includes only a small part of the section in a limited number of cases. However, it offers more information about cell formation, as well as new parameters that allow a differentiation between reactive CD30^+^ cells and neoplastic, clonally expanded CD30^+^ cells in cHL. These changes are visible and quantifiable. Table 2 compares the advantages (+) and limitations (-) of conventional 2D histological sections and 3D depiction.

**Table 2:** Advantages and limitations of 2D and 3D depiction.

	2D	3D
**Cell surface**	-	+
**Cell diameter**	-	+
**Cell shape**	-	+
**Nuclear shape**	+/-	+
**Cell networks**	-	+
**Protrusions**	+/-	+
**Interaction/cell contact**	-	+
**Large sections**	+	-

### Absolute volumes of CD30^+^ cells

High amounts of tumour infiltrated tissue in cHL usually seems to be associated with unfavourable prognosis. By now, measurements of tumor infiltration are performed in 2D, using 2μm thick sections neglecting surface and volume. Our results quantify cytoplasm masses subdivided in total volumes, networks and single cells (Fig 3)

### CD30 expression in adenoids and lymphadenitis is similar

CD30 expression is reported to be expressed on mainly B-cells in inflammatory diseases [27,28], including adenoids of young children (Fig 2). They are reported to be mononuclear and stimulated B-immunoblasts and are located in germinal centres or extra follicular [14,29].

Fig 3(A) indicates similar volume of cytoplasm of CD30^+^ cells in AD compared to LAD. An explanation could be that both tissues contain lymphoid tissue that exists naturally in the body. However, these tissues originate from different organs and differ in the anatomical structure as lymph nodes have a capsule and a subdivision into paracortex, cortex and medulla, whereas adenoids are lymphoepithelial tissue belonging to mucosal-associated lymphoid tissue. Both support the immune defence and respond to pathogens entering the body. It should be noted that lymph nodes are spread throughout the body, whereas adenoids occur in children’s aches and regress in adolescence. They are exposed to infections or allergens that enter via the aerodigestive tract which can become chronic and force adenoidectomy.

In contrast, depending on the region, lymph nodes are exposed to different antigens which can be a viral or bacterial infection in the cervical area, a wound of the upper or lower extremities. We found that the amount of CD30+ cytoplasm is similar in AD and LAD (Fig 3(A)) and that the cell sizes are in average comparable (Fig 3(C)). However, in LAD the sizes of cells showed a higher standard deviation (Fig 3(C)). As multiple reasons lead to lymphadenopathy and subsequent lymph node excision, the diagnose LAD results in various diseases and ends up in variable histologic appearance. We propose that this leads to the inhomogeneous intensity of CD30 staining in our samples and the differences in size of the activated CD30^+^ cells. Adenoids show about the same amount of proliferating cells but the cell sizes seem to be more homogeneous than in lymphadenitis (Fig 3(C)). On may speculate that this might be a result of the similar pathogenes of adenoids. We assume that therefore the cell sizes are more homogeneous. The 3D visualisation of CD30^+^ cells outlines the differences in size without involvement of molecular techniques. It has become evident in one of our recent investigations, that gene expression of CD30^+^ cells in tonsils shows similarities to the expression patterns of RS-cells. Interestingly, genes of various sorted B-cell subsets differed significantly. For example, genes for MYC, the key factor for proliferation, differentiation, and apoptosis are increased in CD30^+^ reactive cells, as well as genes important for the interaction between B- and T-cells, known for HRS-cells [29]. Attention should be drawn to recently published work of Sperling et al. who proved that chronic CD30 signaling of activated B-cells in mice increases the risk of B-cell lymphomagenesis [30]. Therefore, CD30^+^ cells may be precursors of HRS-cells under special conditions.

### Networks and cell connections dominate in NScHL

Light microscopic examination of thin histological sections suggested that CD30^+^ cells show protrusions and constrictions of different sizes, important for their interactions with surrounding cells. Only a 3D depiction of tumour cells can definitely confirm connections and protrusions of cells or cell groups by rotating objects in three axes.

Especially, in NScHL it can be suggested, that intercellular networks, are supporting tumour development and expansion [14,31]. However, this feature has rarely been shown in HL cell lines in 3D depiction but our data for CD30^+^ cytoplasm in human tissue sections matches these assumptions.

Our results confirm that up to 75% of the malignant cells are organized in networks which seems to be characteristic for NScHL (Fig 3(B)). Since we prove that network formations are less common and particularly smaller in reactive entities, we propose that the exchange of information between malignant cells by communicating protrusions may help to modulate the microenvironment according the tumours requirements. Our work initiates the possibility for further evaluation of these networks. For example, studies reported on a variant of NScHL in which synctical growth pattern with HRS-cell aggregates dominates the histopathological pattern in 2D. This subtype called “Synctical Variant NScHL” is likely to be at high risk and is related to a more aggressive clinical course, and a diminished overall survival [32,33].

Cross-linking of the CD30^+^ cells of this subtype is likely to be even enhanced visible in 3D, wherefore 3D network analysis could support diagnostic differentiation especially in contentious cases.

### Cell size and protrusions increase in MCcHL

In our investigation single cells were predominantly found in MCcHL especially when infected with EBV (Fig 3(B), 64% of total volume of cytoplasm)

Considering the mass of CD30^+^ cytoplasm, the total amount in MCcHL is only slightly higher than under reactive conditions (Fig 3(A)), but malignant transformation comes along with an increase of cell size compared to the CD30^+^ reactive counterparts (Fig 3(C)). Our results are consistent with previous research on development of HRS-cells by incomplete cytokinesis and re-fusion of daughter cells [34], as well as increased gene expression causing proliferation [35]. Single cell analysis resulted in small sized cells in NScHL which leads to the assumption that HRS-cells tend to cluster and form networks after gaining multinuclearity since a big cell size comes along with significantly less clonal growth [36].

In thick sections, especially cases of MCcHL, cells displayed homogenous, strong expression of CD30 compared to small reactive CD30^+^ cells (Table 1) correlating with a study that proved that larger cell size comes along with higher intensity of CD30/CD15 phenotype in cell lines using FACS. Interestingly, Janz et al. reported about small cells in different HRS-cell lines which show not only higher protein synthesis, but also advanced nuclear activating transcription factor (ATF3) which is involved in cellular stress and damage [37], indicating different functions of different sized cells. It would be of interest if genetic imbalances between small and big cells become manifest in 3D cell architecture to draw interference from morphology to genetic attributes.

It was reported by Jong et al. that especially in immunodeficiency settings, overlapping of morphologic features of Diffuse-large B-cell Lymphomas, cHL and T-cell and histiocyte-rich large B-cell lymphoma is challenging precluding unequivocal classification [38]. If further techniques provide recognition patterns among different diseases as we found for reactive and malignant entities, this data might support diagnostics in challenging cases.

Furthermore, a special volume of CD30^+^ cells could imply better responding to modern therapy using Brentuximab Vedotin (BV), which has enormously improved therapeutic options [39]. This drug conjugate interacts with CD30^+^ benign and malignant cells by internalising its endotoxin into the cell resulting in specific cell lysis. Therefore, especially large numbers of CD30^+^ cells or large cell sizes, infiltrating the tissue, could respond better to this medication. Since the mechanism of anti-tumor drugs is only partly understood, the histology of the tumorous tissue during or after therapy, using 3D cell visualisation could reveal unknown interactions between the drugs and the target cells [40–42].

Especially in case of co-infection with EBV, CD30^+^ cell cytoplasm increases (Fig 3(C)). although the low number of data in not statistical. The role of EBV infection has extensively been studied and recently been reviewed as it is associated with several diseases including lymphoproliferative disorders, especially cHL [43]. Pavlovic et al. reported about increased numbers of CD8+ T-cells and FOXP3+ T-reg cells in EBV+ individuals indicating its effects on the surrounding microenvironment of cHL [44].

Furthermore, there is not only difference in chemokine and cytokine expressions between EBV- and EBV+ infected cells, but also support for HRS-cells to survive and proliferate due to activation of signalling pathways [35]. Expression of the latent proteins LMP1, LMP2A and EBNA1, cause activation of signalling pathways as e.g. NFκ -B, JAK/STAT, PI3-K/Akt which affects HRS-cell survival or T-cell immunity [45]. Our values for the larger cell size in EBV+ MCcHL may reflect these changes and should be further be evaluated as EBV infection is supposed to take an active part in the pathogenesis of cHL [43,45].

Concerning cell protrusions, Hansen et al. proved in cell lines using 3D techniques that these in vitro branch-like cell extensions are used for the exchange of cytokines and chemokines produced by the tumor cells [31]. Especially in MCcHL we detected these protrusions frequently (Table 2). It was reported that HRS-cells are able to modulate surrounding fibroblasts by mediating vesicles which are not only changing fibroblast phenotype but also their secretome into cancer associated fibroblasts (CAFs), emphasising their malignant potential [31,46]. These extracellular vesicles are too small to be visualized by our method whereas cell protrusions and strangulations can clearly be detected especially in malignant cells. In LAD and AD they occur less often which may also result in smaller networks.

To understand the function and role of the protrusions in cell communication, further studies that combine 3D visualisation and molecular techniques might be useful.

It has to be considered, that there is no comparable data about cHL cell volume so far, as no equivalent approach has been reported yet. Since our data correlate with existing 2D data we see potential to investigate further information about different cell populations using this method.

### Limitations

This study is limited by the relatively small cohort of cases. Additional analysis should be performed according to the established method using different stainings since we are addressing CD30, independently from other cell subsets. CD30 characterizes the cytoplasm of the cell. Nuclei are not taken into account in the calculations. Furthermore, it should be pointed out, that we did not include cell particles that were neither determined as individual cells nor belonging to networks. These fragments were excluded in our approach, since we characterized cells by specific criteria and under equal and reproducible conditions. Further studies could take these cell fragments into account by applying new definitions. Additionally, since no data existed to compare our absolute values with, we consider the shift in volumes when comparing reactive and malignant disorders for most important.

## Conclusion

Summarising the results, 3D cell visualisation of thick paraffin tissue sections offers a spectrum of new scientific and diagnostic opportunities. In comparison to 2D, 3D histological techniques provide new parameters, like cell surface, cytoplasmic volume and network affiliation. This investigation is the first attempt to compare CD30^+^ cells in lymphadenitis and cHL to point out characteristic cell formation in 3D cell systems. We consider this investigation as a pilot project and new approach to answer functional questions at the level of 3D morphological findings. As our visual and quantitative results correlate with existing literature, we propose this technique as an enhancement of diagnostic tools using standard histological sections which should be further investigated to prove its benefit. Moreover, our work contributes to the idea of integrating 3D data into computing systems and enhancing digital research and diagnostics.

## Supporting information

S1 Table Data on volumes, networks and single cells. (XLS)
